# Mild cognitive impairment and quality of life in the oldest old: a closer look

**DOI:** 10.1007/s11136-020-02425-5

**Published:** 2020-01-28

**Authors:** Felix S. Hussenoeder, Ines Conrad, Susanne Roehr, Angela Fuchs, Michael Pentzek, Horst Bickel, Edelgard Moesch, Siegfried Weyerer, Jochen Werle, Birgitt Wiese, Silke Mamone, Christian Brettschneider, Kathrin Heser, Luca Kleineidam, Hanna Kaduszkiewicz, Marion Eisele, Wolfgang Maier, Michael Wagner, Martin Scherer, Hans-Helmut König, Steffi G. Riedel-Heller

**Affiliations:** 1 Institute of Social Medicine, Occupational Health and Public Health, University of Leipzig, Ph.-Rosenthal-Str. 55, 04103 Leipzig, Germany; 2 Institute of General Practice, Medical Faculty, Heinrich-Heine-University Düsseldorf, Düsseldorf, Germany; 3 Department of Psychiatry, Technical University of Munich, Munich, Germany; 4 Central Institute of Mental Health, Medical Faculty Mannheim/Heidelberg University, Mannheim, Germany; 5 Institute for General Practice, Hanover Medical School, Hanover, Germany; 6 Department of Health Economics and Health Services Research, University Medical Centre Hamburg-Eppendorf, Hamburg, Germany; 7 Clinic for Neurodegenerative Diseases and Geriatric Psychiatry, University Hospital of Bonn, Bonn, Germany; 8 Institute of General Practice, Medical Faculty, Kiel University, Kiel, Germany; 9 Department of Primary Medical Care, Center for Psychosocial Medicine, University Medical Center Hamburg-Eppendorf, Hamburg, Germany; 10 Clinic for Psychiatry and Psychotherapy, University Hospital of Bonn, Bonn, Germany; 11 German Center for Neurodegenerative Diseases (DZNE), Bonn, Germany

**Keywords:** MCI, Quality of life, Older people, WHOQOL-OLD

## Abstract

**Purpose:**

Mild cognitive impairment (MCI) is a widespread phenomenon, especially affecting older individuals. We will analyze in how far MCI affects different facets of quality of life (QOL).

**Methods:**

We used a sample of 903 participants (110 with MCI) from the fifth follow-up of the German Study on Ageing, Cognition, and Dementia in Primary Care Patients (AgeCoDe), a prospective longitudinal study, to analyze the effects of MCI on different facets of the WHOQOL-OLD. We controlled for age, gender, marital status, education, living situation, daily living skills, and the ability to walk, see, and hear.

**Results:**

Univariate analyses showed that individuals with MCI exhibited lower QOL with regard to the facets autonomy; past, present, and future activities; social participation; and intimacy, but less fears related to death and dying. No significant difference was shown with regard to the facet sensory abilities. In multivariate analyses controlling for age, gender, marital status, education, living situation, daily living skills, and the ability to walk, see and hear, MCI-status was significantly associated with QOL in the facet autonomy.

**Conclusion:**

Effects of MCI go beyond cognition and significantly impact the lives of those affected. Further research and practice will benefit from utilizing specific facets of QOL rather than a total score.

## Introduction

Age-related cognitive decline is a widespread phenomenon, and there is an adjusted overall prevalence of 16% (effect size, 95% CI 12–20%), increasing with age, for mild cognitive impairment (MCI) [10]. In the light of demographic change and an aging society, cases of MCI are likely to rise. It is therefore crucial to understand the subjective implications of MCI for those affected. In the long run, this will help to develop suitable interventions and support.

Quality of life (QOL) is a key concept for understanding the subjective dimension of MCI and the impact it has on those affected. Most researchers agree on a multidimensional concept including physical, psychological, and social aspects as well as those related to daily life activities [6, 25]. In addition, over the lifespan, different aspects become relevant for QOL. Since our interest is in the oldest old, we assessed QOL with the WHOQOL-OLD, an instrument that specifically addresses domains that are relevant for individuals older than 60 years [4]: (1) sensory impairments and in how far they affect daily life as well as the ability to communicate with others (sensory abilities); (2) the amount of autonomy, independent decision taking, and ability to influence one’s future (autonomy); (3) received appreciation and felt satisfaction for accomplishments in life as well as a general future outlook (past, present, and future activities); (4) level of activity and possibilities to participate (social participation); (5) fears and attitudes related to death and dying (death and dying); and (6) possibilities to experience love and affection (intimacy).

Research comparing quality of life (QOL) of individuals with and without MCI is rare and inconsistent. While some studies report no difference between the two groups [19, 23], others report lower QOL for people diagnosed with MCI in almost all areas [28, 31]. Especially individuals who are aware of their MCI diagnosis seem to have reduced QOL independent of impairment severity [27]. Unfortunately, most studies refer to highly selective samples from memory clinics or nursing homes [19] or are focused on health-related QOL [14, 17, 22]. Only few studies are representative for the general population [15]. In addition, research on MCI is often centered around the idea of MCI as a risk factor for subsequent dementia, rather than on the direct effects of MCI on the individuals [2, 24, 34].

## Aims of the study

In this study, we want to investigate how MCI is associated with QOL. We will therefore (1) analyze the differences between individuals with and without MCI diagnosis in QOL in general and with regard to all six facets of QOL, i.e., sensory abilities; autonomy; past, present, and future activities; social participation; fears related to death and dying; and intimacy, and (2) analyze the predictive effect of MCI on the scores of QOL facets controlling for well-established variables like age and marital status.

## Methods

### Study design and sample

We used data derived from the German Study on Ageing, Cognition, and Dementia in Primary Care Patients (AgeCoDe), a prospective longitudinal study on the early detection of MCI and dementia in general practices that was conducted as a collaboration of six study centers—Hamburg, Bonn, Duesseldorf, Leipzig, Mannheim, Munich. Baseline assessment took place in 2003–2004, and participants were reassessed in follow-ups every 18 months until 2013. Participants were recruited based on the following inclusion criteria: (1) aged 75 and over, (2) absence of dementia, (3) at least one GP contact within the last year. Patients were excluded, if (4) GP consultations were home visits only, (5) patients lived in a nursing home, (6) GPs diagnosed a severe illness which they would deem fatal within 3 months, (7) patients were deaf, blind, lacked sufficient proficiency in the German language, or lacked the ability to provide informed consent.

Out of a randomly selected, cross-sectional sample of *N* = 6619 GP patients, a total of *N* = 3327 eligible persons consented to participate and were assessed at baseline through structured clinical interviews. The design of the study has been described in detail elsewhere [18].

**Fig. 1:**
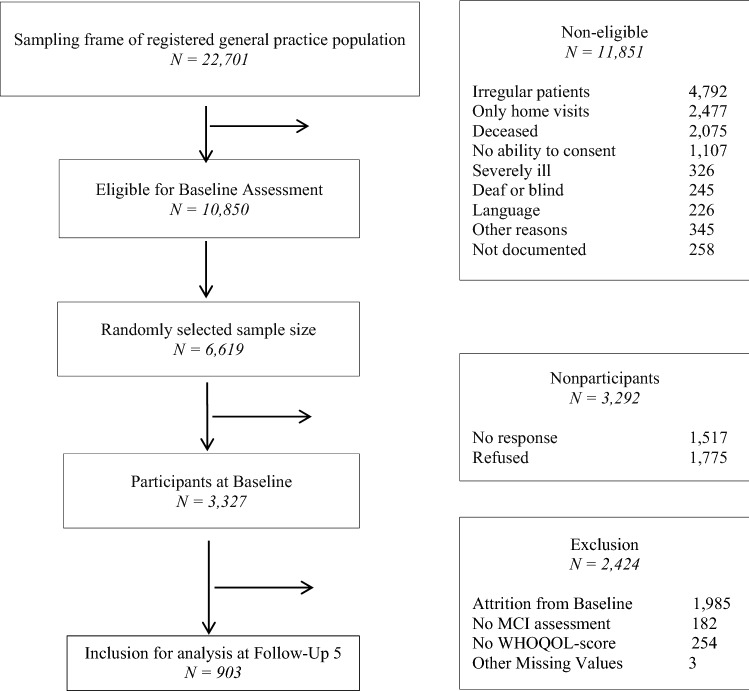
Process of sample selection

For the present study, we utilized data from Follow-up 5, collected between 10/2010 and 11/2012, as here QOL had been assessed. 2424 out of 3327 participants from baseline assessment were not part of Follow-up 5: 1985 related to study attrition mainly due to death, 182 related to unclear MCI-status, 254 related to incomplete WHOQOL measurement, and three related to missing control variables. The final sample comprised a total of 903 participants. A detailed depiction of the sample selection process is found in Fig. 1.

### Assessment

Structured clinical interviews were conducted by trained psychologists and physicians at participants’ homes. The interviewers assessed control variables age, gender, marital status, education, and current living situation. Research shows that higher age, better education, being married, being male, and living with a partner can be associated with better quality of life [13, 21, 30]. In addition, the ability to hear, see and walk, and the ability to function in daily life—which are all positively associated with quality of life [7, 9, 16, 26]—were assessed, and cognitive tests were conducted.

### Cognitive assessment and MCI-status

MCI was diagnosed according to consensus criteria proposed by the International Working Group on Mild Cognitive Impairment [32]. They include the following: (1) absence of dementia according to DSM-IV or ICD-10, (2) evidence of cognitive decline: self-rating or informant report and impairment on objective cognitive tasks and/or evidence of decline over time on objective cognitive tasks, and (3) preserved baseline activities of daily living or only minimal impairment in complex instrumental functions. Dementia according to DSM-IV was excluded with structured interviews (SIDAM) [35]. The criterion of subjective cognitive complaints was fulfilled when the question on subjective memory impairment was positively answered. The SIDAM neuropsychological test battery was used to assess objective cognitive decline. Impairment in all four cognitive domains was defined as test performance of more than 1 SD below the main value for age- and education-specific norms. The functional activities were assessed with the SIDAM-ADL Scale. Participants with only one or no impairments in the 14 items of the SIDAM-ADL Scale were regarded as functionally unimpaired.

### Quality of life

Quality of life was assessed using the WHOQOL-OLD, a test that had been specifically designed to assess the subjective QOL of adults over the age of 60. It includes six facets, with four items each: sensory abilities; autonomy; past, present, and future activities (assesses received appreciation and felt satisfaction for accomplishments in life as well as a general future outlook); social participation; fears related to death and dying; and intimacy [4, 5]. Items can be answered from 1, representing absolute disagreement, to 5, i.e., absolute agreement. During analysis inverse items were recorded before an average was calculated for every facet. This facet average was then multiplied by four, resulting in a score between four and 20 which was then transformed into a score between 0 and 100. Higher scores represent better QOL.

### Instrumental abilities

The ability to carry out instrumental activities of daily living (IADL) with the Lawton-and-Brody IADL scale [1]. The scale includes abilities related to using the telephone, shopping, food preparation, housekeeping, laundry, mode of transportation, and responsibility for own medications and finances.

### Statistical analyses

Independent *t* tests and the Mann–Whitney test (death and dying) were used to analyze QOL differences between participants with and without MCI diagnosis.

We used Stata 16 and multiple linear regressions to analyze the effect of MCI on QOL facets sensory abilities; autonomy; past, present, and future activities; social participation; and intimacy controlling for age, gender, marital status, living situation, ability to carry out instrumental activities of daily living (IADL), and the ability to walk, see and hear. Since there was right-censoring in the observed range of death and dying, we used Tobit regression for that specific facet. The Huber–White sandwich estimator [8] was applied in all regressions to obtain a robust variance estimate that adjusts for within-cluster correlation with regard to study centers.

## Results

### Descriptive characteristics

After excluding participants without scores on the main variables, our dataset contained 903 individuals with 603 females (66.8%) and 110 (12.2%) with MCI. Table 1 shows the general characteristics of the study population. The MCI group was slightly older, less likely to be married, slightly better educated, exhibited lower IADL scores and was more likely to have hearing problems and considerable or severe walking impairments.

**Table 1:** General characteristics of the study population

	Total group (N = 903)	No MCI (N = 793)	MCI (N = 110)
Age	86.5 (3.1)	86.3 (2.9)	87.9 (3.8)***
Female	603 (66.8%)	530 (66.8%)	73 (66.4%) n.s
Marital status			n.s
Single	59 (6.5%)	51 (6.4%)	8 (7.3%)
Married	279 (30.9%)	250 (31.5%)	29 (26.4%)
Divorced	49 (5.4%)	38 (4.8%)	11 (10.0%)
Widowed	516 (57.1%)	454 (57.3%)	62 (56.4%)
Education^a^			***
Low	489 (54.2%)	460 (58.8%)	29 (26.4%)
Medium	292 (32.3%)	228 (28.8%)	64(58.2%)
High	122 (13.5%)	105 (13.2%)	17 (15.5%)
Living situation			n.s
Alone	475 (52.6%)	417 (52.6%)	58 (52.7%)
With partner	283 (31.3%)	252 (31.8%)	31 (28.2%)
With relatives or others	55 (6.1%)	46 (5.8%)	9 (8.2%)
Assisted, retirement/nursing home	95 (10.5%)	83 (10.0%)	12 (10.8%)
MCI	110 (12.2%)	0 (0%)	110 (100%)
Instrumental Activities (IADL)^b^	6.6 (1.7)	6.7 (1.6)	5.7 (2.2)***
Problems walking			**
No impairment	370 (41.0%)	336 (42.4%)	34 (30.9%)
Mild impairment	437 (48.4%)	382 (48.2%)	55 (50.0%)
Considerable/severe impairment	96 (10.7%)	75 (9.5%)	21 (19.1%)
Problems seeing			n.s
No impairment	699 (77.4%)	608 (76.7%)	91 (82.7%)
Mild impairment	151 (16.7%)	135 (17.0%)	16 (14.5%)
Considerable/severe impairment	53 (5.9%)	50 (6.3%)	3 (2.7%)
Problems hearing			*
No impairment	464 (51.4%)	418 (52.7%)	46 (41.8%)
Mild impairment	417 (46.2%)	358 (45.1%)	59 (53.6%)
Considerable/severe impairment	22 (2.4%)	17 (2.2%)	5 (4.5%)

Continuous variables are given as mean (standard deviation), and *p* values refer to independent *t* tests; categorical variables are displayed as numbers (percentages), and *p* values refer to Chi-square tests

*MCI* mild cognitive impairment

*p ≤ 0.05; **p ≤ 0.01; ***p ≤ 0.001

^a^Education classification according to the Comparative Analysis of Social Mobility in Industrial Nations (CASMIN)

^b^Representative score of the German population = 6.7 (SD: 1.7) (Conrad et al. 2016)

### Group comparisons: quality of life in non-MCI vs. MCI

Table 2 shows comparisons between the MCI and the non-MCI groups for all six facets of QOL, indicating significant differences for all facets besides sensory abilities. While participants with MCI in general exhibited reduced QOL, they experienced less fears related to death and dying.

**Table 2:** Differences in QOL (WHOQOL-OLD) between individuals without and with MCI

Dimension	No MCI (*N* = 793)	MCI (*N* = 110)	Test-statistic
Total	68.81 (.43)	66.23 (1.17)	***t* (901) = 2.10, *p* = .038**
Sensory abilities	68.09 (.75)	65.18 (2.05)	*t* (897) = 1.36, *p* = .175
Autonomy	68.89 (.60)	63.21 (1.73)	***t* (899) = 3.25, *p* = .001**
Past, present, and future activities	69.04 (.53)	65.39 (1.51)	***t* (893) = 2.41, *p* = .016**
Social participation	68.34 (.61)	64.03 (1.60)	***t* (895) = 2.50, *p* = .013**
Death and dying	68.10 (.86)	73.36 (2.16)	U = 37,562, *z* = − 2.17, *p* = .030*
Intimacy	70.58 (.74)	66.28 (1.85)	***t* (886) = 2.03, *p* = .043**

*MCI* mild cognitive impairment

*Since criteria for the independent *t* test were not fulfilled, the Mann–Whitney test was used

### MCI as a predictor of QOL

Table 3 shows the regression analysis with MCI as a predictor of QOL facets and control variables. Results show that age, gender, marital status, and seeing and hearing abilities showed only little relevance for the prediction of QOL facets (besides the expectable prediction of sensory abilities). Education significantly predicted two facets (autonomy, past, present, and future activities), IADL three (autonomy, past, present, and future activities, social participation), and walking ability predicted three facets (sensory abilities, past, present, and future activities, social participation). MCI-status exhibited a significant, negative impact on autonomy. Since our predictors explain almost no variance in the outcome of death and dying, the effects of specific predictors will not be interpreted.

**Table 3:** Impact of MCI, sociodemographic and health variables on facets of QOL (unstandardized regression coefficients)

Dimension	Total (*N* = 903)	Sensory abilities (*N* = 899)	Autonomy (*N* = 901)	Past, present, future activities (*N* = 895)	Social participation (*N* = 897)	Death and dying^a^ (*N* = 899)	Intimacy (*N* = 888)
Constant	22.51	55.74	29.45	24.86	17.00	4.72	0.36
MCI	− 1.50	− 1.84	− 4.23*	− 2.10	− 0.95	5.25*	− 4.32
Age	0.43*	0.20	0.33	0.40	0.44	0.64	0.61*
Gender	1.00	− 0.19	− 1.38	0.94	2.39*	7.98**	− 3.13
Marital status (vs. single)							
Married	8.25*	3.60	2.20	8.95	8.19	10.71*	17.10*
Divorced	2.02	− 0.34	4.20	0.98	3.10	− 0.08	4.01
Widowed	5.73*	1.42	2.61	6.29	4.98	10.43**	9.43*
Education (vs. low)							
Medium	0.97	2.13	1.42	− 0.14	− 0.11	0.19	1.91
High	1.44	2.04	5.31*	1.79*	0.97	− 3.61***	1.35
Living situation (vs. alone)							
With partner	− 0.06	− 0.90	− 2.07	− 0.69	− 2.34	− 1.08	6.33
With relatives or others	3.68*	2.60	1.70	3.98	2.24	8.61*	6.39
Assisted, retirement/nursing home	0.72	3.95	− 0.67	1.14	− 0.014	− 1.33	1.31
Daily living skills (IADL)	1.10*	0.68	1.87*	0.98*	2.34**	0.09	0.74
Walking (vs. no impairment)							
Mild impairment	− 4.07**	− 4.03*	− 4.63	− 3.88***	− 9.17***	− 3.24	− 0.79
Considerable/severe	− 4.92*	− 2.87	− 7.29	− 6.88**	− 16.45**	2.51	1.90
Seeing (vs. no impairment)							
Mild impairment	− 2.93*	− 8.06**	− 0.88	− 2.47	− 2.22	− 3.16	− 1.41
Considerable/severe	− 4.48*	− 22.66***	− 3.01	0.71	− 6.67*	2.71	3.18
Hearing (vs. no impairment)							
Mild impairment	-3.66**	− 14.64***	− 1.99	− 1.88	− 2.56*	− 0.29	− 0.54
Considerable/severe	− 5.06*	− 25.96***	− 0.35	0.62	2.64	− 6.01	− 1.45
*R*^2^	0.15	0.26	0.13	0.10	0.27	0.01	0.10

The Huber–White sandwich estimator (Froot 1989) was used in all regressions to obtain a robust variance estimate that adjusts for within-cluster correlation with regard to study centers

*MCI* mild cognitive impairment

*p ≤ 0.05; **p ≤ 0.01; ***p ≤ 0.001

^a^Tobit regression

## Discussion

In our sample, 12.2% of participants were diagnosed with MCI. This relatively low percentage, compared to other current studies [10], is a consequence of the application of strict criteria for MCI diagnosis [32]. Our univariate comparison of participants with and without MCI showed significant differences for QOL for the facets autonomy; past, present, and future activities; social participation; death and dying; and intimacy. With the exception of death and dying, the non-MCI group constantly showed higher levels of QOL. This could point to the fact that in case of MCI, existential fears are to some extent replaced by more proximate fears and worries related to daily living. The regression analysis, including multiple control variables, confirmed MCI diagnosis as a significant predictor of impaired QOL in the facet autonomy.

Our results from the univariate analysis show that MCI has a tendency to affect QOL in a negative way. This matches with other studies where MCI had a negative association with psychological QOL [20] and with QOL measured via subject and informant ratings [29]. The fact that there was no difference between MCI and non-MCI groups in terms of QOL with regard to sensory abilities is most likely a consequence of the strong impact of seeing and hearing abilities on this specific facet. In the multivariate analysis, we can find an impact of MCI-status on the facet autonomy. This makes sense, since the decline of cognitive abilities, and the fear of further deterioration, directly affects individuals’ ability to live independently and take own decisions. In addition, some researchers emphasize the importance of promoting autonomy in order to increase health-related QOL of people with MCI [3]. Since MCI is far less severe than dementia and more heterogenic in outcome [32], impairments are smaller and less visible to others. Therefore, MCI may not so much affect social interactions as reflected in the facets intimacy and social participation. The fact that MCI was not associated with social participation can be seen as a potential resource for those affected by MCI, since frequency of engagement in social activities is linked to a lower risk of progression from mild to severe forms of cognitive impairment [11] and social activities are connected to reduced dementia risk [12].

## Conclusion

Results indicate that, especially, autonomy is associated with MCI. In the light of high prevalence of MCI among older people, these results have implications for the management of MCI. For example, participants may benefit from interventions to boost autonomy and/or cope with decreasing independence.

From a methodological point of view, our results show that in order to understand the burden of MCI on the older population, a differentiated approach, using specific facets rather than a total score, is highly recommendable.

From the perspective of research, interactions between facets are of great interest as well as the causal mechanisms and the neuronal, behavioral and psychological processes that link MCI to QOL. Furthermore, future research may differentiate between the effects of amnestic vs. non-amnestic MCI and take into account the severity of impairment.

Reduced QOL, especially over longer periods of time, may have multiple consequences for individuals on a psychological, physiological, behavioral, and social level. Clearly, more research is needed in this area.

## Limitations

While this study has several advantages, e.g., the comprehensive assessment of MCI and QOL, and a well-described cohort of individuals in late life, our research also has certain limitations. For living situation and the ability to walk, see and hear, answering options were merged for analysis due to the small amount of participants attached to specific options. Further research would benefit from a more differentiated approach to MCI with regard to type, amnestic vs. non-amnestic, and degree of severity. In addition, our analysis is cross sectional which limits our ability to make a causal claim to the findings of the study.
